# Hydrological Classification, a Practical Tool for Mangrove Restoration

**DOI:** 10.1371/journal.pone.0150302

**Published:** 2016-03-23

**Authors:** Anne F. Van Loon, Bram Te Brake, Marjolein H. J. Van Huijgevoort, Roel Dijksma

**Affiliations:** 1 School of Geography, Earth and Environmental Sciences, University of Birmingham, Birmingham, United Kingdom; 2 The Birmingham Institute of Forest Research (BIFoR), University of Birmingham, Birmingham, United Kingdom; 3 Eijkelkamp Soil & Water, Giesbeek, the Netherlands; 4 Hydrology and Quantitative Water Management Group, Wageningen University, Wageningen, the Netherlands; Agharkar Research Institute, INDIA

## Abstract

Mangrove restoration projects, aimed at restoring important values of mangrove forests after degradation, often fail because hydrological conditions are disregarded. We present a simple, but robust methodology to determine hydrological suitability for mangrove species, which can guide restoration practice. In 15 natural and 8 disturbed sites (i.e. disused shrimp ponds) in three case study regions in south-east Asia, water levels were measured and vegetation species composition was determined. Using an existing hydrological classification for mangroves, sites were classified into hydrological classes, based on duration of inundation, and vegetation classes, based on occurrence of mangrove species. For the natural sites hydrological and vegetation classes were similar, showing clear distribution of mangrove species from wet to dry sites. Application of the classification to disturbed sites showed that in some locations hydrological conditions had been restored enough for mangrove vegetation to establish, in some locations hydrological conditions were suitable for various mangrove species but vegetation had not established naturally, and in some locations hydrological conditions were too wet for any mangrove species (natural or planted) to grow. We quantified the effect that removal of obstructions such as dams would have on the hydrology and found that failure of planting at one site could have been prevented. The hydrological classification needs relatively little data, i.e. water levels for a period of only one lunar tidal cycle without additional measurements, and uncertainties in the measurements and analysis are relatively small. For the study locations, the application of the hydrological classification gave important information about how to restore the hydrology to suitable conditions to improve natural regeneration or to plant mangrove species, which could not have been obtained by estimating elevation only. Based on this research a number of recommendations are given to improve the effectiveness of mangrove restoration projects.

## Introduction

Mangrove forests are valuable coastal ecosystems in tropical coastal regions around the world [1–3]. Due to increased pressures in these regions, such as logging, aquaculture and coastal development, mangrove forests are declining worldwide [4–8]. In many countries the value of mangroves for coastal protection, ecosystem functioning and supporting livelihoods of coastal communities has been recognised and restoration projects have been set up. Unfortunately, many of these restoration projects have been unsuccessful [9–12].

Reasons for failure of mangrove restoration projects are myriad, ranging from natural to social processes. However, some of the natural processes that determine species distribution patterns in natural mangrove forests (i.e. propagule dispersal, light conditions, [13–15]) are not important in restoration sites. For example, propagules can be supplied and light is often abundant. Successful restoration often comes down to site conditions suitable for mangrove survival [9], of which the most important are salinity, soil conditions and hydrology. Hydrology is often overlooked in mangrove restoration projects [16], making it an important reason for failure (as shown in research by [9, 10, 17–22]). For example, mangrove seedlings are planted in the mudflat zone, which is too wet for mangrove growth, or in shrimp ponds mangrove vegetation fails to recover after abandonment due to impaired flow conditions [23]. Restoring the hydrology of impounded mangrove areas has proven to lead to successful restoration in Florida [24], Costa Rica, the Philippines [25], and Thailand [26].

One of the reasons that hydrology is often not taken into account is that it is not easy to quantify. A recent United Nations Environment Programme Report [27] made a plea for using more scientific knowledge in restoration projects. One of the approaches that was recommended is Ecological Mangrove Restoration (EMR), a ‘community-based restoration practice that uses several physical and ecological principles to support natural recolonisation’ [9, 27]. In this approach focus is shifted from planting of seedlings to physical site preparation. According to the EMR method, there are five key principles for successful mangrove restoration (after [9, 27]):

1. Understanding the individual species ecology at a potential restoration site;
2. Understanding normal hydrological patterns controlling seedling establishment and successful growth of mangrove species;
3. Assessing current environmental obstacles and modifications of the original mangrove habitat that currently prevent establishment and succession;
4. Designing a restoration program to restore appropriate hydrology and address conditions preventing natural colonisation of mangrove propagules and plant establishment;
5. Only planting propagules or seedlings after steps 1-4 have been taken and if natural recruitment is not sufficient to provide the quantity of successfully established seedlings, the soil stabilisation or rate of growth necessary for the project.

Sometimes an additional step is added between step 3 and 4, which accounts for funding, manpower, and land ownership issues [28]. The EMR approach has been widely used in restoration practise because it combines scientific knowledge with experience of local management and communities.

In the EMR steps, hydrology is specifically mentioned as an important factor [9, 22, 29, 30]. One of the pressing questions is how hydrology should be taken into account in practical applications of step 2, 3 and 4 of the EMR method for mangrove restoration. Research has shown that inundation duration and frequency are important hydrological factors in the distribution of mangrove species and that these inundation characteristics are related to elevation [31–33]. The EMR approach, therefore, advises to take a tide chart of a nearby tidal station, estimate the topography of a healthy mangrove stand and mimic that topography in the restoration site [34]. A hydrological classification, linking topography and hydrology to mangrove species, can be a useful tool in this procedure, but this has never been tested scientifically.

In this paper we want to test methods for taking hydrology into account within the steps of the EMR approach. Our overall aim is to provide a tool for improving hydrological understanding in mangrove restoration projects. Our specific objectives are to test the usefulness of a hydrological classification to i) understand normal hydrological patterns in mangrove forests (EMR step 2); ii) assess hydrological modifications in restoration sites (EMR step 3); and iii) design the restoration of appropriate hydrology (EMR step 4). We also compared our method based on measurement of water levels with the method of using elevation only. During the research we kept in mind the requirements of mangrove restoration projects, i.e. the need for simple methods that can be applied with minimal equipment and minimal technical knowledge and skills. Finally, we describe the results of a sensitivity analysis on the methodology, evaluate uncertainties and give recommendations.

## Hydrological classification for mangroves

Currently, the classic hydrological mangrove classification of Watson (1928) [31] is still applied and recommended [15, 35–38]. From extensive research in Malaysia, [31] found that the species growing in the mangrove forests of Malaysia can be grouped into 5 classes based on three variables: tidal regime, elevation, and flooding frequency (see Table 1). Areas in class 1 are inundated too often for mangrove species to survive, resulting in mudflats devoid of vegetation. In class 2 only pioneer species like *Avicennia sp.* and *Sonneratia sp.* can establish. Class 3 is the most diverse class, with hydrological conditions suitable for groups of species including *Rhizophora sp.*, *Ceriops sp.* and *Bruguiera sp*‥ Class 4 is inundated by the tides only rarely and therefore allows other species groups to enter the mangrove vegetation composition, e.g. *Lumnitzera sp.*, *Bruguiera sp.* and *Acrosticum sp*‥ The highest class, which is almost never inundated, is only suitable for mangrove species such as *Phoenix paludosa* Roxb.

**Table 1:** Watson’s classification derived for Malaysian mangrove systems, from [31].

class	tidal regime	elevation	flooding frequency
-	-	[m + admirality datum]	[times per month]
1	all	<2.44	56–62
2	medium	2.44–3.35	45–56
3	normal	3.35–3.96	20–45
4	spring	3.96–4.57	2–20
5	equinoctial	>4.57	<2

Despite the wide application of Watson’s classification outside Malaysia, [39] pointed out some disadvantages of this classification for a more general application. The most important drawback is that Watson’s classification is developed for regions with a regular tidal regime and a regular elevation profile. [39] found that due to an irregular tidal regime and micro-topography the inundation characteristics of a mangrove forest showed much higher spatial variability than expected. Inundation duration was, for example, longer than expected based on elevation and flooding frequency because water was ponding behind natural levees. Additionally, the tidal regime in the case study site in Vietnam [39] was a mix of diurnal and semi-diurnal tides, making the variable ‘flooding frequency’ in Table 1 unsuitable as a proxy for hydrological conditions.

Therefore, [39, 40] and [41] proposed some changes to the original Watson classification to make it more suitable to irregular tides and elevation (see Table 2). The most important changes include:

- splitting the most diverse class 3 into class 2* and class 3, to reflect the higher sensitivity of mangrove species to hydrological conditions around class 3;
- omitting the variable ‘tidal regime’, because it is too vague and not useful in situations with irregular tides;
- using ‘duration of inundation’ instead of ‘frequency of inundation’, to increase usability in situations with irregular elevation profiles and irregular tides;
- introducing two ways of measuring duration of inundation, because [39] found that these are both important to determine the correct vegetation class.

**Table 2:** Adapted hydrological classification including common southeast Asian mangrove species groups, from [40], based on [31] and [39]. Elevation (in italics) is included only as proxy for inundation characteristics for mangrove regions with regular tidal regime and regular elevation profile.

class	*elevation*	duration of inundation	duration of inundation	species
-	*[cm + MSL]*	[min per day]	[min per inundation]	-
1	*<0*	>800	>600	none
2	*0–50*	400–800	450–600	*A. alba* Blume, *Sonneratia sp.*
2*	*50–100*	250–400	200–450	*Avicennia sp.*, *Rhizophora sp.*, *Bruguiera sp.*
3	*100–150*	150–250	100–200	*Rhizophora sp.*, *Ceriops sp.*, *Bruguiera sp.*
4	*150–210*	10–150	50–100	*Lumnitzera sp.*, *Bruguiera sp.*, *Acrosticum aureum* L.
5	*>210*	<10	<50	*Ceriops sp.*, *Phoenix paludosa* Roxb.

Note that a variable ‘elevation’ is still included in Table 2, but that [39, 40] and [41] do not recommend its use. [39] mention that elevation can only be used in mangrove regions with regular tidal regime and regular elevation profile, where no measurements of water levels can be done and accurate measurements of elevation exist. The adapted classification of Table 2 has up to now only been used in natural mangrove areas and not in mangrove restoration projects.

## Study area description

**Fig 1:**
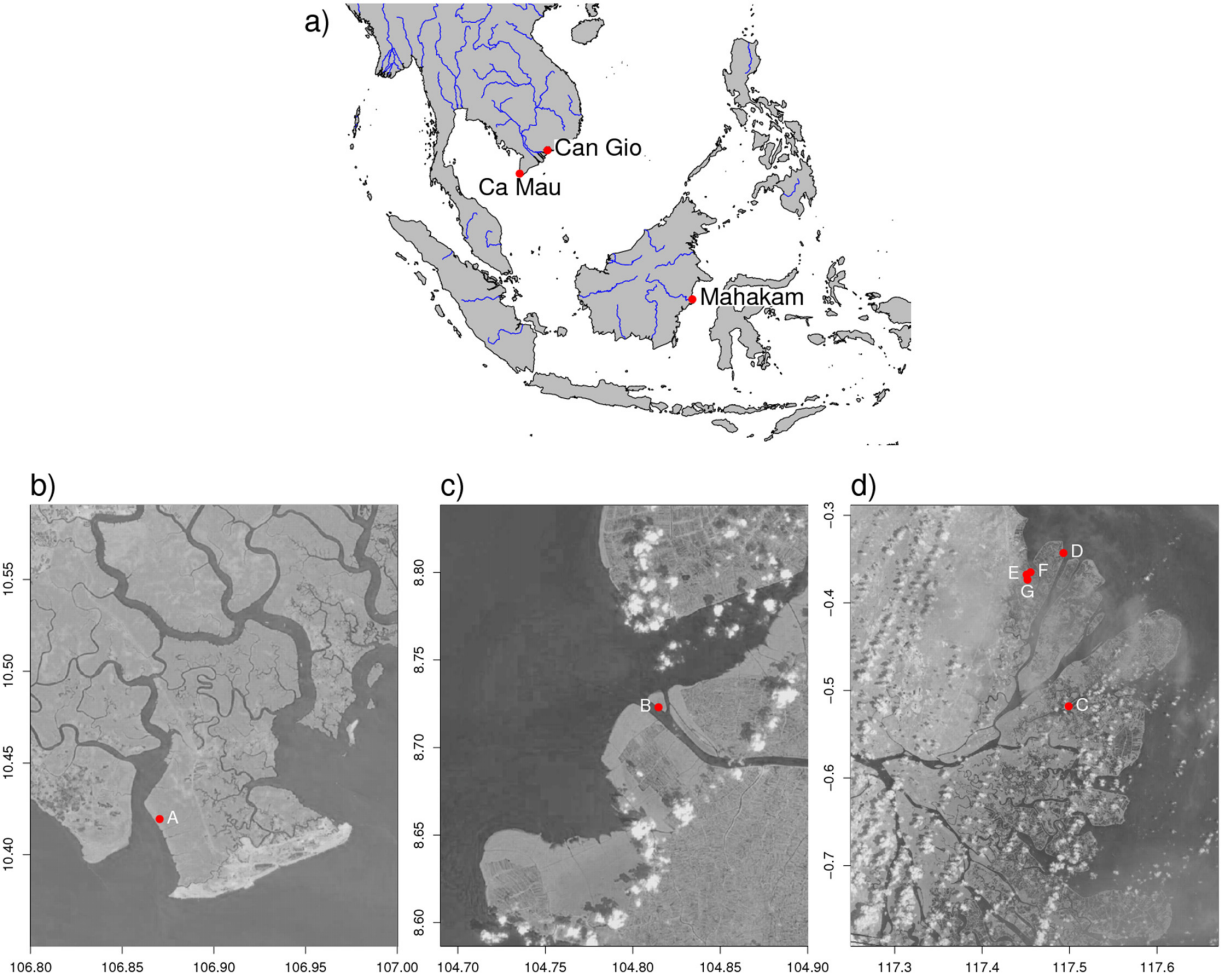
Location of study regions and measurement locations. a) study regions in south-east Asia (made with Natural Earth), b) measurement locations in Can Gio—Vietnam, c) Ca Mau—Vietnam, and d) Mahakam—Indonesia. Landsat data available from the U.S. Geological Survey.

The fieldwork for this study was done in three mangrove regions in south-east Asia: Can Gio and Ca Mau in Vietnam and Mahakam in Indonesia (Fig 1a). In this section, we give a description of the geography, climate, tidal regime, and vegetation of each of the three regions and indicate the measurement locations that were chosen within these regions.

### Can Gio—Vietnam

The Can Gio UNESCO Man and the Biosphere Reserve (Fig 1a) is a mangrove area of 750 km^2^ in Vietnam. It is part of the Saigon-Dong Nai river delta located in the Ho Chi Minh City Province [46]. One of the largest rivers in the delta is the Dong Tranh river, which discharges into the South China Sea [39]. The tidal regime of the South China Sea near Can Gio is irregular semi-diurnal, with a maximum amplitude of 3.3–4.1 m [47]. The climate is tropical with a high and constant temperature (Table 3) and a high precipitation varying between a wet season (May–October) and a dry season (November–April). Can Gio is a low-lying delta area; almost the entire area has an elevation between 0 and 2 m above sea level [48]. It consists of a complex network of rivers, channels, creeks and gullies (Fig 1b) and topography is dynamic. The soil of Can Gio is composed of river clay and silt deposited by the Saigon-Dong Nai river [49] and marine sands from the South China Sea [50]. The rivers in Can Gio show a pronounced variation in discharge between the wet and dry season, despite the regulating effect of reservoirs upstream.

**Table 3:** Location characteristics for the case study sites in Vietnam and Indonesia. For the hydrologically disturbed sites years since abandonment or plantation are indicated.

location	name	average P [mm/yr]	average T [C]	tidal range [m]	natural / disturbed
A	Can Gio (Vietnam)	1300-1400^a^	25.8^a^	2.3—4.1	natural + semi-natural plantation (25 yrs)
B	Ca Mau (Vietnam)	2000-2400^b^	26.5-27.3^b^	0.7—1.7	natural
C	Mahakam (Indonesia)	1704^c^	27.6^c^	1.5—2.2	natural
D	Mahakam (Indonesia)	1704	27.6	1.5—2.2	natural
E	Mahakam (Indonesia)	1675^d^	27.4^d^	1.5—2.2	abandoned shrimp pond (3 yrs)
F	Mahakam (Indonesia)	1675	27.4	1.5—2.2	restored shrimp pond (10 yrs)
G	Mahakam (Indonesia)	1675	27.4	1.5—2.2	restored shrimp pond (3 yrs)

^a^ from [42]

^b^from [43]

^c^for 2009, from [44]

^d^for 2011, from [45]

Part of the mangrove forest of Can Gio that was destroyed in the Second Indochina war (1963–1974) has been replanted with *Rhizophora apiculata* Blume in 1978 [51]. Since then, natural regeneration in open spaces and along the edges resulted in mixed natural vegetation. Especially lightning gaps and storm disturbance greatly increased biodiversity [52]. Can Gio was declared as a Biosphere Reserve in 2000, mainly to protect the mangroves from being converted into shrimp farms [52].

**Fig 2:**
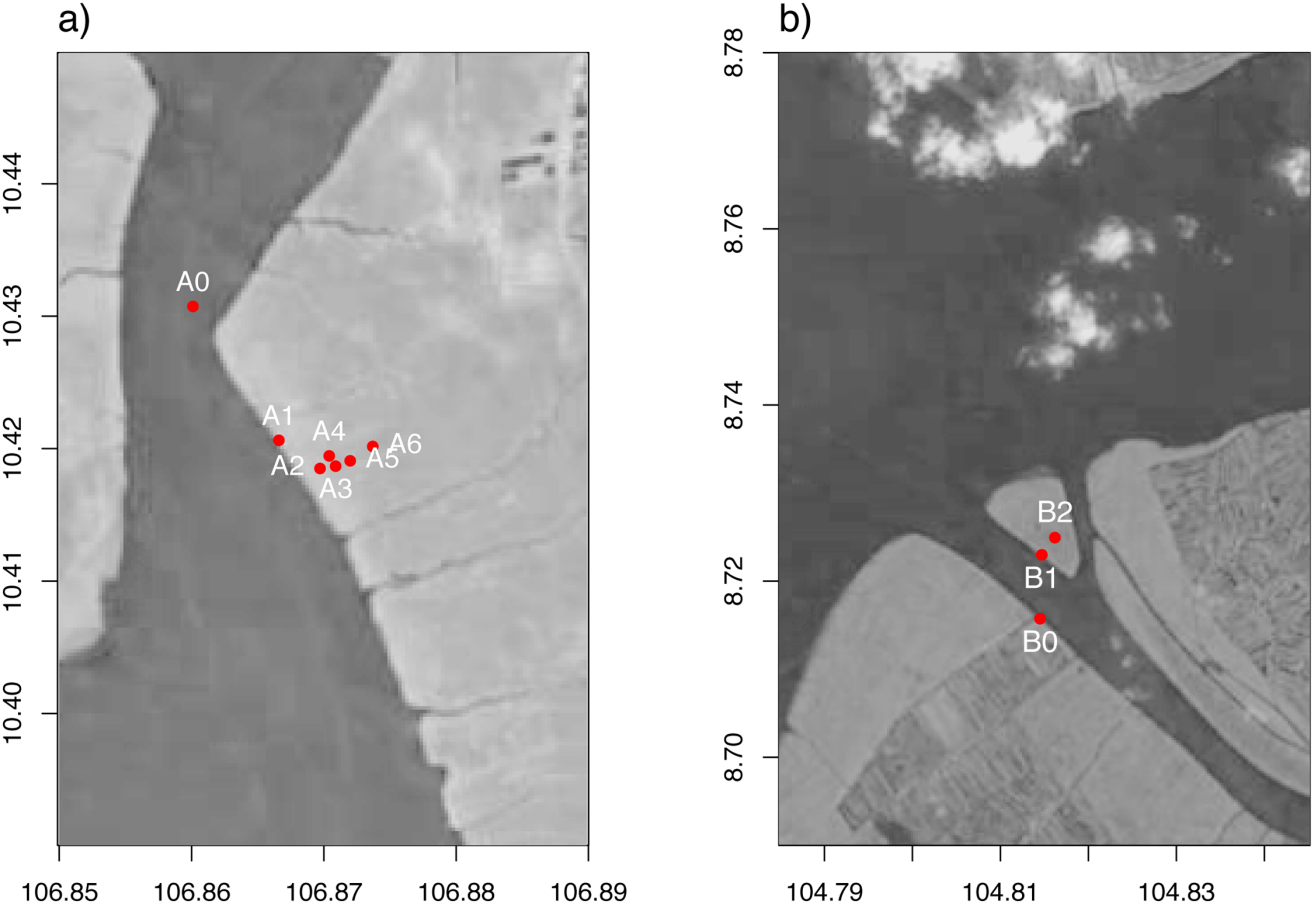
Location of measurement sites in Vietnam. a) Can Gio, b) Ca Mau. Measurement sites in open water (A0, B0) and in the mangrove forest (see Table 4). Landsat data available from the U.S. Geological Survey.

Measurement location A is a transect perpendicular to the Dong Tranh river, with a total length of 700 m (Fig 2a). Vegetation shows a clear distribution from *Avicennia alba* Blume close to the river to *Rhizophora apiculata* Blume further inland, with zones with higher biodiversity in between (Table 4).

**Table 4:** Site characteristics for all measurement sites in Vietnam and Indonesia. Site elevation was determined using the method described in Section “Data and Methods” and vegetation is given in order of dominance of the species at the measurement site. *A.* = *Avicennia*, *R.* = *Rhizophora*.

site	measurement period	elevation [cm + MSL]	distance to main channel/sea [m]	vegetation
A1	3-Mar-2007/25-May-2007	12	18	*A. alba* Blume
A2	17-Mar-2007/14-Apr-2007	92	205	*Avicennia sp.*, *Rhizophora sp.*, *Ceriops decandra* (Griff.) Dong Hou
A3	3-Mar-2007/25-May-2007	104	330	*R. apiculata* Blume, *A. marina* (Forsk.) Vierh., *A. officinalis* L.
A4	17-Mar-2007/14-Apr-2007	114	330	*Avicennia sp.*, *R. apiculata* Blume, *Ceriops decandra* (Griff.) Dong Hou
A5	3-Mar-2007/25-May-2007	116	450	*Avicennia sp.*, *R. apiculata* Blume, *Ceriops decandra* (Griff.) Dong Hou
A6	3-Mar-2007/25-May-2007	121	680	*R. apiculata* Blume, *Acrostichum aureum* L.
B1	21-Apr-2007/20-May-2007	46	50	*A. alba* Blume, *Sonneratia alba* J.Smith, *R. apiculata* Blume
B2	21-Apr-2007/20-May-2007	35	255	*R. apiculata* Blume, *Bruguiera parviflora* (Roxb.) Wight & Arn., *A. marina* (Forsk.) Vierh.
C1	02-Nov-2009/05-Dec-2009	98	25	*A. officinalis* L., *R. stylosa* Griff.
C2	08-Nov-2009/05-Dec-2009	67	20	*Bruguiera parviflora* (Roxb.) Wight & Arn., *A. officinalis* L., *R. stylosa* Griff.
C3	08-Nov-2009/05-Dec-2009	-18	10	*R. stylosa* Griff., *A. officinalis* L., *Sonneratia caseolaris* (L.) Engl.
D1	06-Dec-2009/29-Dec-2009	-40	at coast	*Sonneratia alba* J.Smith
D2	06-Dec-2009/29-Dec-2009	-10	35	*Sonneratia alba* J.Smith, *A. officinalis* L.
D3	06-Dec-2009/29-Dec-2009	18	55	*A. officinalis* L., *Bruguiera gymnorhiza* (L.) Lam., *Sonneratia alba* J.Smith
D4	06-Dec-2009/29-Dec-2009	89	100	*Acrostichum aureum* L.
E1	20-Dec-2011/18-Jan-2012	-45	1000	none
E2	20-Dec-2011/18-Jan-2012	-19	1000	none
E3	20-Dec-2011/19-Jan-2012	15	1000	none
F1	20-Dec-2011/17-Jan-2012	-23	670	*Avicennia sp.*, *Rhizophora sp.*
F2	20-Dec-2011/19-Jan-2012	20	670	*Rhizophora sp.*
G1	20-Dec-2011/19-Jan-2012	23	950	*Avicennia sp.*, *Rhizophora sp.*
G2	20-Dec-2011/19-Jan-2012	-41	950	none
G3	20-Dec-2011/19-Jan-2012	16	950	*Rhizophora sp.*

### Ca Mau—Vietnam

Mui Ca Mau National Park is located in the Ca Mau peninsula, the southernmost part of Vietnam (Fig 1a). There are no major rivers discharging into the peninsula, only a channel intersecting the peninsula connecting the South China Sea to the Gulf of Thailand (Fig 1c). This means that the geomorphology of Ca Mau is dominated by wave action instead of fluvial processes. The tidal regime is a combination of a diurnal regime with 0.5–1.0 m amplitude in the Gulf of Thailand and an irregular semi-diurnal regime with 2.5–3.8 m amplitude in the South China Sea. The combination of both tidal regimes and the complex creek system in Ca Mau peninsula causes water interactions that are not fully understood [53]. The climate of Ca Mau is similar to that of Can Gio, with slightly higher temperature and precipitation (Table 3). Soils in the Ca Mau peninsula are highly determined by sediments from the Mekong river, transported by the South China Sea, and forming sandy beach ridges on the coast and deposition of finer sediment more inland. The entire peninsula is a low-lying area, within a 2 m range. Soil texture is clayey or loamy for 95% of the soils [53].

Much of the original vegetation cover was destroyed in the Second Indochina war (1963–1974), but natural regeneration and planting programs led to a partial vegetation recovery with dominance of *Avicennia sp.* and *Rhizophora sp.* [46, 54]. Major socio-economic changes occurred in the Ca Mau area since the 1990s [54]. Intensive rice and shrimp production was made possible by reduction of salinity intrusion [55]. This, in combination with overexploitation of mangrove resources due to population growth, largely contributed to the loss of mangrove forests in Ca Mau. It also caused major changes in drainage patterns and tidal flooding frequency in large parts of the area [53]. In 2003, the Mui Ca Mau National Park was established protecting 42,000 ha on the southwestern tip of the peninsula. The National Park prohibits any anthropogenic activities in core zone (except small-scale fishing in open water) and restricts forest exploitation and aquaculture activities in buffer and transition zones.

Measurement location B is on the Con Ngoi island in the Cua Lon channel (Figs 1c and 2a). This island is located in the strict protection zone of the Mui Ca Mau National Park. The mangrove forest on this recently formed island is completely naturally generated (Table 3) and consists mainly of *Avicennia sp.* and *Rhizophora sp.* combined with some other species (Table 4).

### Mahakam—Indonesia

The Mahakam Delta is located on the east coast of Kalimantan, Indonesia (Fig 1a). It is a fan-shaped mixed tide—fluvial dominated delta system, formed by deposition of sediments from the Mahakam river upstream catchment [56, 57]. The Mahakam branches out before entering the Makassar strait (Fig 1d). The tidal regime of the Makassar strait at the Mahakam coast is irregular semi-diurnal [58] with an amplitude of 0.5–1.7 m [59]. The area has a tropical rainforest climate [56] with a constant temperature of 25.5 degrees Celsius and a precipitation of 2000–3000 mm/year with a pronounced seasonal cycle (Table 3). Contrasting to Can Gio there is little variability in flow and no flood surges occur because big lakes in the catchment dampen the flow. This also results in a lack of natural levees in the Mahakam delta [56].

Over the past decades, large social changes have occurred in the Mahakam delta as a result of mass immigration from other Indonesian islands of people finding work in aquaculture, mining, forestry, and oil and gas exploitation [60, 61]. The mangrove forest of the Mahakam delta was drastically reduced and fragmented during rapid expansion of the aquaculture industry from 1980 to 2000 with up to 55% of the forest area being converted to aquaculture ponds [62]. What was left of the mangroves is a narrow band along the shrimp ponds and colonisation of newly formed sedimentary islands. Since 2002, some restoration of mangroves was initiated, partly because decreased shrimp production in the delta left many shrimp ponds abandoned. Muara Badak, located in the northern section of the Mahakam Delta, for example, has a restoration program set up by the district government that has resulted in a raised awareness for the need of restoration. In some areas natural regeneration occurred, but in others a monoculture of *Rhizophora apiculata* Blume was planted.

**Fig 3:**
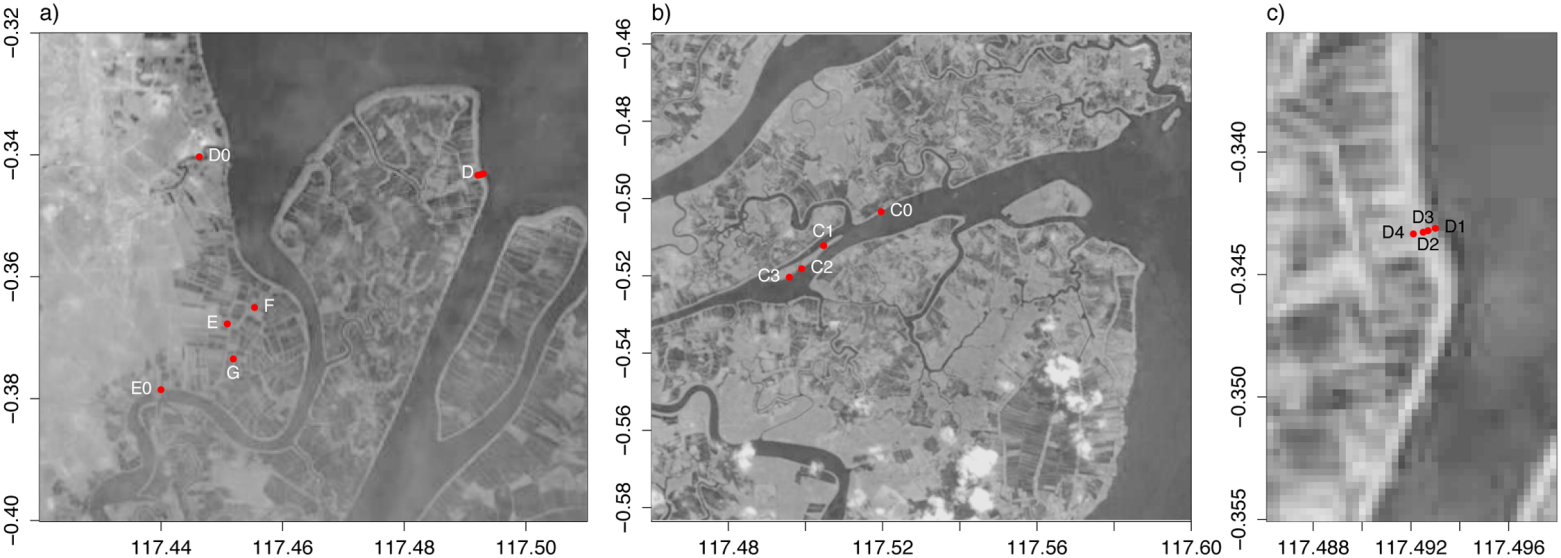
Location of measurement sites in Mahakam—Indonesia. a) measurement sites in open water (D0, E0) and inland (D–G), b) measurement sites of location C, c) measurement sites of location D (see Table 4). Measurement sites in location E, F, and G are too close together to indicate separately. Landsat data available from the U.S. Geological Survey.

Measurements were taken at several locations in the delta focusing on natural (C and D) and hydrologically disturbed (E, F and G) conditions (Fig 1d). Location C is on an elongated island in the river about 9 km inland from the coast (Fig 3a). The vegetation has a natural mix of different species (Table 4). Location D is in the northern part of the delta at the coast (Fig 3b). Measurements were done in a transect of 100 m perpendicular to the coast (Fig 3c). Vegetation shows a distribution from *Sonneratia alba* J.Smith at the coast to *Acrostichum Aureum* L. inland (Table 4).

**Fig 4:**
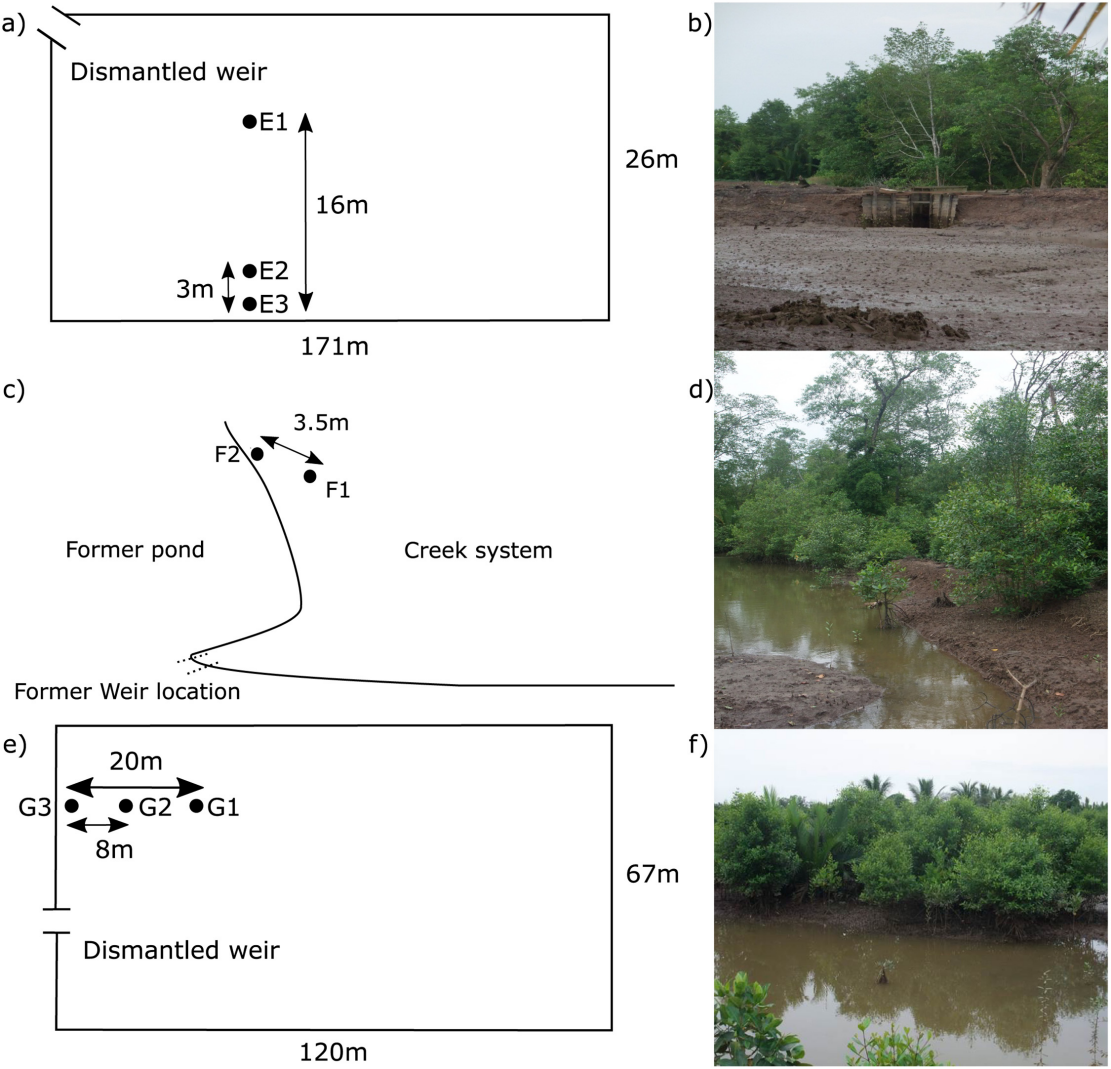
Measurement sites in location E–G in Mahakam—Indonesia. a) measurement setup location E, b) photo location E, c) measurement setup location F, d) photo location F, e) measurement setup location G, f) photo location G.

Locations E, F and G were chosen as examples of restoration sites with disturbed hydrological conditions (Fig 1d and Table 4). Location E is a shrimp pond abandoned since 2008 and with dikes around the pond still present (Fig 4a and 4b). There are some natural *Nypa Fruticans* Wurmb and planted coconut palms around the edges, but no vegetation inside the pond. Site F is a shrimp pond in the process of restoration with 10 years of new growth in and around the pond (Fig 4c and 4d). Water moves freely here and measurement sites were located just outside the original pond. Site G is a shrimp pond undergoing restoration; abandoned in approximately 2005 and replanted 3 years later (Fig 4e and 4f). Water can enter the pond freely, but dikes are still intact due to neighbouring active shrimp ponds. Measurement sites in location E, F, and G were chosen close together to measure spatial variability within the shrimp ponds.

## Data and Methods

The data used in this research have been collected during field campaigns in several years. In Can Gio and Ca Mau, Vietnam, measurements were done in 2007. In Mahakam, Indonesia, measurements were conducted in 2009 (locations C and D) and in 2011–2012 (locations E, F and G; Table 4). In each campaign, water levels were measured and vegetation inventories were made. The measurements were kept to a minimum to mimic the practical requirements of restoration projects. In this section, we describe the methodology used to obtain and analyse the data.

### Field Methods

**Fig 5:**
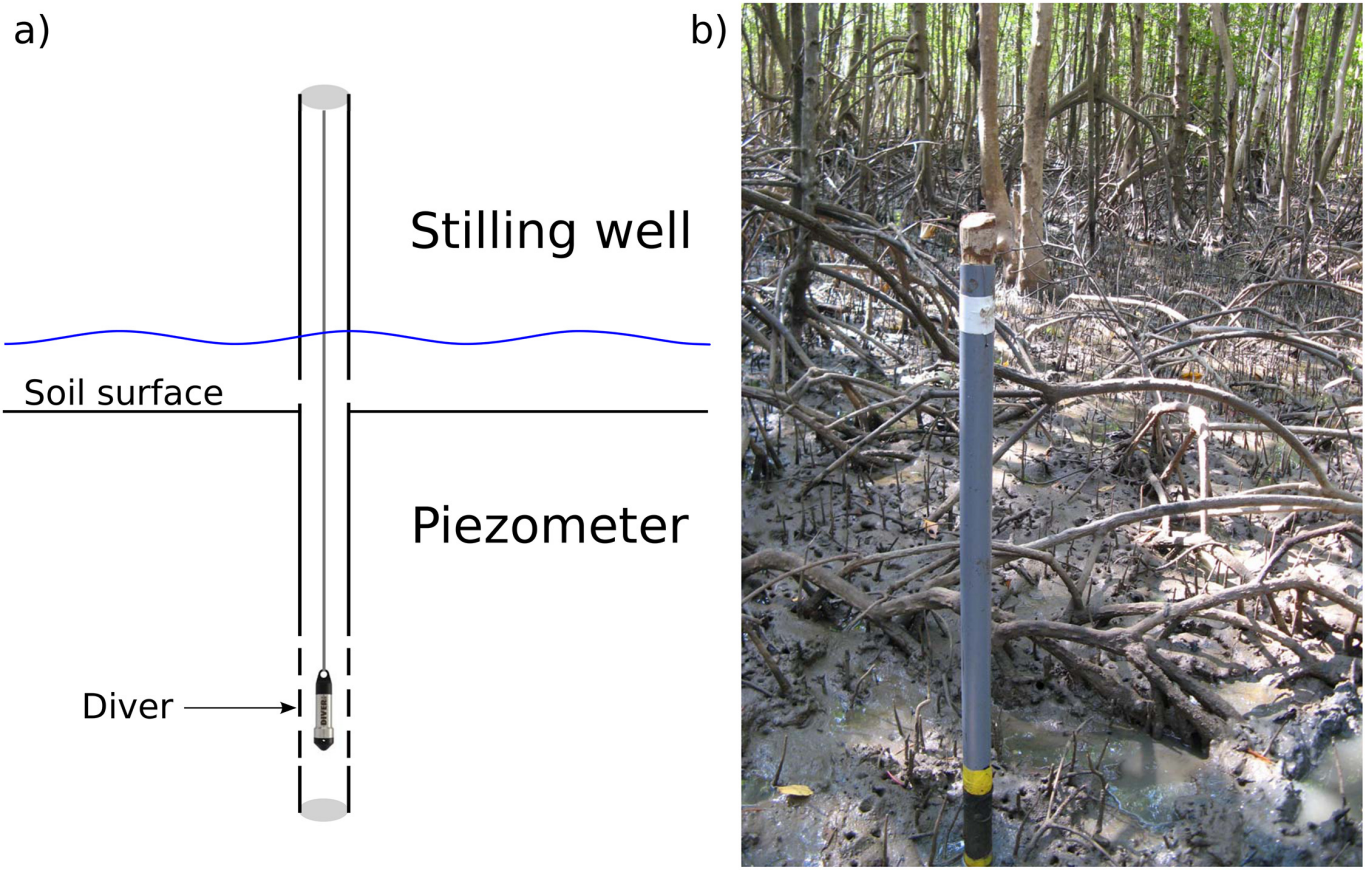
Water level measurement set up. a) diver installed in tube that serves as stilling well above soil surface and as piezometer below soil surface, b) installation in the field.

Water levels were measured with Diver Water Level Loggers (hereafter called divers [63]), installed in tubes that served both as piezometer (below soil surface level) and stilling well (above soil surface level; Fig 5). The tubes were installed with ca. one meter below and one meter above surface level. Because divers are absolute pressure transducers, measuring combined atmospheric pressure and water pressure, atmospheric pressure was measured by divers installed in nearby buildings or cabins (one per location).

Water levels were measured in open water and in the mangrove forest (Figs 2 and 3). The open water sites were located in the river system close to the forest sites. Measurement sites in the forest were set up in a transect in locations with a clear mangrove zonation (location A and D) and had a more scattered distribution elsewhere. The objective for the selection of sites was to obtain a range of characteristics (e.g. elevation, vegetation, history of human disturbance). For the natural locations (A–D), diversity in vegetation and elevation was desired. For the disturbed locations (E–G), we additionally took into account the history of the location (restoration or not, time since restoration, etc.).

Water levels were measured with a measurement frequency of 5 minutes. The measurement period was aimed to be around 30 days or a multitude of 30 days, in order to measure a complete lunar tidal cycle. This was not achieved at all sites (Table 4).

The vegetation inventories were carried out in a small zone around each water level measurement site. No standard area was used for these vegetation inventories, but a representative area was chosen. Mangrove vegetation was identified and documented using a classification handbook [64] and with help of local experts. Dominance, age and health condition of species were estimated, but no measurements were taken on the individual trees. The presence and species of propagules (seedlings) and young saplings (up to 1 m in height) was noted.

All other characteristics of the sites, such as soil surface level and elevation, were not measured but determined from the water level data.

### Data Analysis

**Fig 6:**
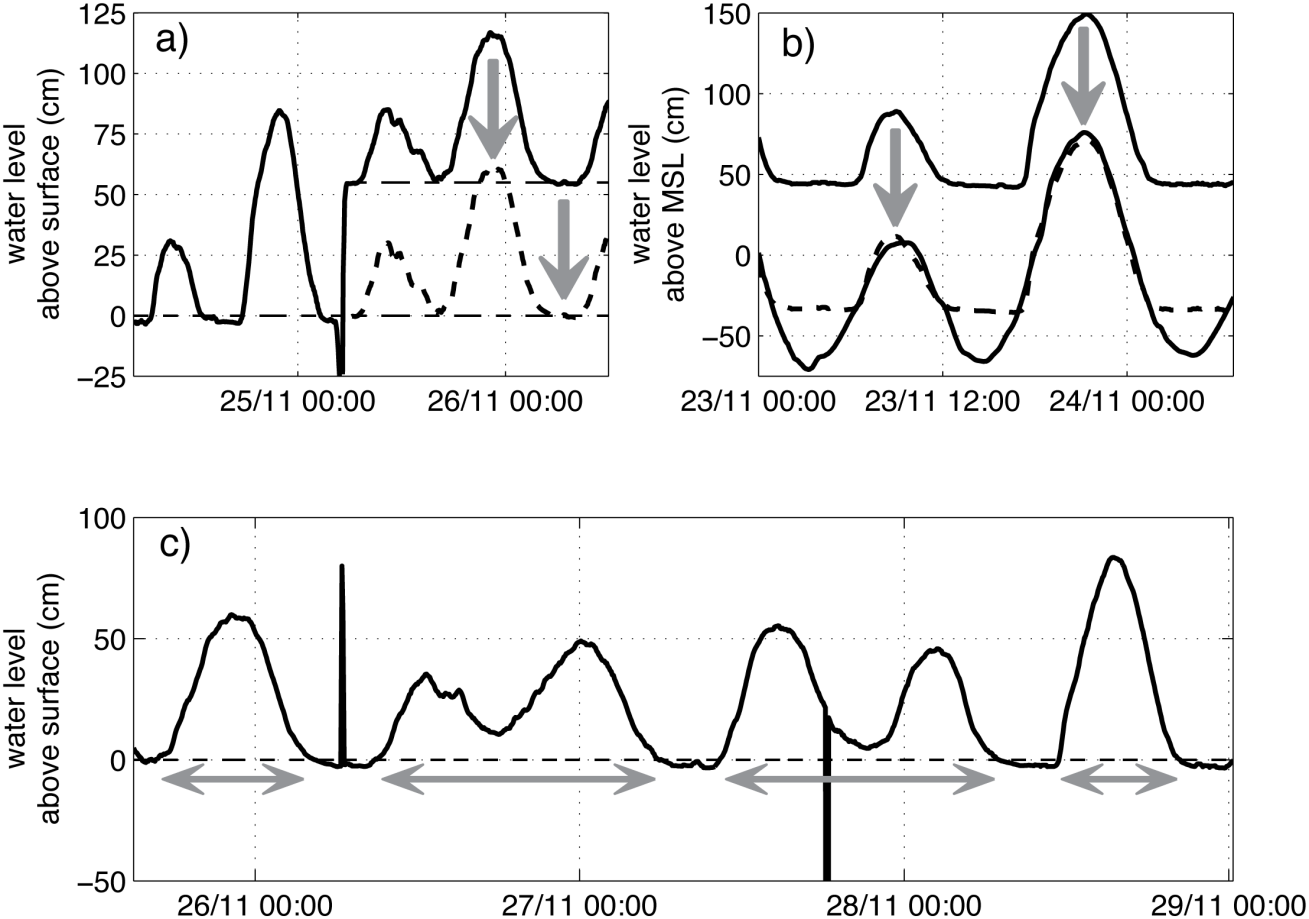
Example of time series of water levels to show the methodology used for time series analysis. a) estimation of surface level from nod point and correction of changes in reference level, b) estimation of elevation with respect to mean sea level, c) correction of measurement errors and calculation of duration of inundation. The horizontal dashed lines represent estimated soil surface level.

Since the observed water levels represent water both on and under the surface, we had to estimate surface level for each site. We used a method to derive surface level directly from the water level data. At ebb, measured water levels drop following the tide until surface level is reached, after which reduced subsurface flow velocities result in a lower falling rate of water levels. The resulting nod point in the time series represents surface level and was set to 0 (Fig 6a—left side).

The nod point was not constant over the entire period of observation because of changes in the height of the diver after replacing the diver when reading the data, and changes in surface level around the piezometer due to soil processes and animal and human disturbance (Fig 6a—right side). Because of these variations in time, the method of determining surface level from water level observations was found to be much more accurate than measuring the length of the cable of the diver and the height of the top of the tube above surface level, even when this was done repeatedly [40].

By counting the measurement intervals with water levels above soil surface level, inundation durations and number of inundations were obtained. The minimum duration of inundation and dry-fall was set at 15 minutes to remove measurement errors, outliers, and small fluctuations in water levels (Fig 6c). [65] and [30] found that duration of inundation is most important in determining mangrove species distribution. We applied two variables related to duration of inundation (i.e. average duration per inundation and per day) that are relevant in irregular tidal regimes [39]. Based on these two variables, sites were then classified into hydrological classes using Table 2.

For comparison of our results with elevation-based methods, we had to estimate elevation for the measurement sites. Because methods such as laser levelling proved to be highly inaccurate [39, 40], we here used the estimated surface levels and the water level time series to determine the elevation of each of the forest sites with regard to mean sea level. Mean sea level was taken as the mean water level measured by the open water diver. Under the assumption that water levels are level in the open water and throughout the mangrove forest at maximum high tide (following [66]), the time series of water levels of the forest measurement sites were overlaid onto those of their respective open water diver (Fig 6b). From the difference between the surface level at each site and the average water level of the open water site, the elevation of the sites was determined (Table 4).

The results of the vegetation inventory around the measurement site were converted into an ‘expected class’ using Table 2. The dominant species was most important, but we also looked at presence of young saplings, which better represent the suitability of the present hydrological regime, because since the establishment of the mature trees sedimentation and erosion might have resulted in changed hydrological conditions. In some sites replanting with *Rhizophora sp.* was done, e.g. in A3, A6, F2 and G3 (Table 4). These sites were classified in 2*, 3, or 4 on the basis of other species present at the measurement site. In some sites in location C and D, unique classes could not be determined because vegetation was mixed. This resulted in combined expected classes.

## Results and Discussion

### Understanding normal hydrological patterns in mangrove forests

The final hydrological classification for all sites in Vietnam and Indonesia is provided in Table 5 and compared with the expected class based on vegetation. In the classification of the sites in Can Gio (A1–A6) we see concurrent changes in hydrological conditions and vegetation from wetter to drier conditions. The average class matches well with expected class based on vegetation; only site A6 is one class off. In Ca Mau, there is agreement between the hydrological and expected class for site B2, but not for site B1, which can be explained by the fact that sedimentation takes place at high rates on the recently-formed small natural island and natural colonisation and succession are still ongoing.

**Table 5:** Water level observations, resulting hydrological classes, and vegetation classes for all measurement sites in Vietnam and Indonesia.

location	site	duration of inundation	resulting class	duration of inundation	resulting class	average class	expected class (based on vegetation)
		*min per day*	-	*min per inundation*	-	-	-
Can Gio	A1	802	1	520	2	1-2	2
	A2	296	2*	220	2*	2*	2*
	A3	254	2*	219	2*	2*	2*
	A4	149	4	209	2*	3	3
	A5	118	4	207	2*	3	3
	A6	100	4	275	2*	3	4
Ca Mau	B1	131	4	221	2*	3	2*
	B2	280	2*	214	2*	2*	2*
Mahakam	C1	141	4	225	2*	3	2*
	C2	257	2*	196	3	2*-3	2*/3
	C3	869	1	477	2	1-2	2*
	D1	911	1	504	2	1-2	2
	D2	608	2	307	2*	2-2*	2/2*
	D3	528	2	231	2*	2-2*	2*/3
	D4	76	4	148	3	3-4	4
	E1	1060	1	656	1	1	-
	E2	800	2	406	2*	2-2*	-
	E3	560	2	318	2*	2-2*	-
	F1	846	1	408	2*	2	2*
	F2	554	2	266	2*	2-2*	3
	G1	527	2	278	2*	2-2*	2*
	G2	1102	1	614	1	1	-
	G3	642	2	296	2*	2-2*	3

Since the classification was developed in Vietnam [39], there was a need to validate it in natural sites in the Mahakam Delta in Indonesia, before it could be applied to restoration sites in that region. Sites D1–D4 show a general agreement between the vegetation and the hydrology (Table 5), with a clear distribution of mangrove species from wet to dry. Expected classes are only slightly higher than observed hydrological classes, indicating that conditions might have changed since the establishment of the mangroves or that species can cope with wetter conditions than expected. Sites C1–C3 have more variation, with the expected class lower than the observed hydrological class in C1 and higher in C3.

From Table 5 we cannot determine which of the two hydrological variables (i.e. duration of inundation in *min per day* or in *min per inundation*) used for classification is dominant; we see that both are important to match the hydrological class with the expected class based on vegetation. Duration of inundation in *min per day* seems dominant in the higher sites (e.g. A6, D4) and *min per inundation* in low-lying sites (e.g. A1, C3, D1). Together, these two variables provide the range in hydrological conditions needed to explain the differences in mangrove vegetation.

### Assessing hydrological modifications in restoration sites

Given the promising results in the natural sites, we applied the classification to hydrologically disturbed restoration sites in Mahakam. At sites E1 to E3, no vegetation was present at the time of measurement (Table 4). However, the hydrological class (Table 5) shows that, except for site E1, the hydrological conditions are in fact suitable for many mangrove species (Table 2). At sites F1 and F2, the vegetation present reflects slightly drier conditions than observed. At site G2, no vegetation was present at the time of measurement, but *Rhizophora sp.* had been planted before. The hydrological conditions at this site are not suitable for mangrove species like *Rhizophora sp.* (too wet). At sites G1 and G3 the planted *Rhizophora sp.* did survive and *Avicennia sp.* came in naturally. The hydrological conditions of G1 and G3 are suitable for a range of mangrove species (Tables 2 and 5).

**Fig 7:**
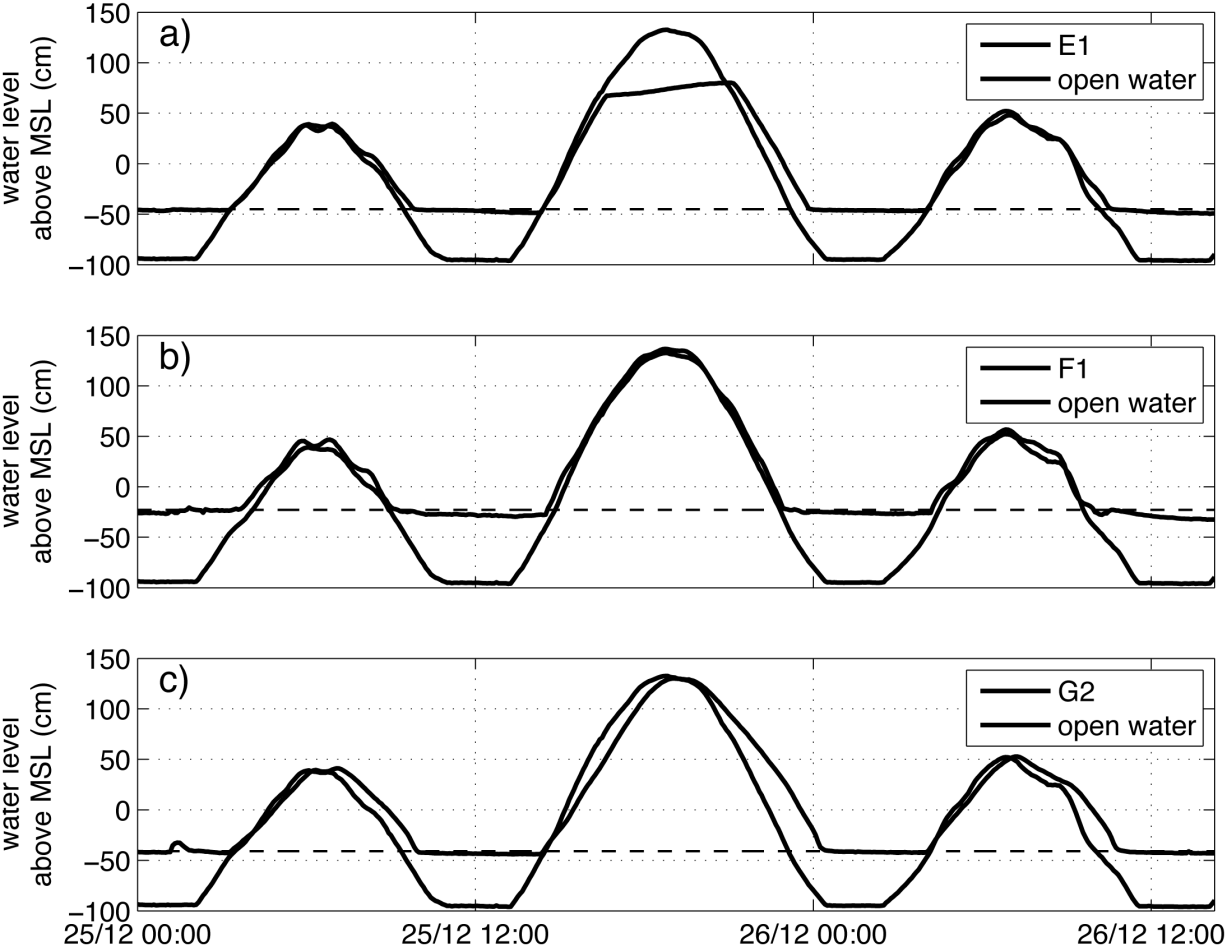
Time series of water levels in three hydrologically disturbed sites in Mahakam (E1, F1, G2) and their respective open water site.

To evaluate the reasons for certain hydrological and vegetation conditions and possible mismatches between them in the restoration sites, we studied water level time series of some selected sites (E1, F1 and G2) in more detail and compared them with open water levels (Fig 7).

At site E1, we see a clear capping at the higher high tides; water levels never reach above 80 cm, which is up to 50 cm lower than the tidal maximum for the measurement period. The reason for this capping is unclear, but might be related to obstruction of dikes. It does, however, not influence inundation duration. The obstruction of dikes also causes tailing (i.e. higher water levels than expected during ebb), which does result in longer inundations, on average 2.6% longer in *min per day* and 10% longer in *min per inundation* compared to a situation with free outflow (Table 6). This effect of tailing is reflected in the resulting class. Site E1 has a lower class in reality (i.e. wetter conditions) than expected from the open water levels (Table 6).

**Table 6:** Difference between hydrological characteristics and class calculated from observed water levels in forest sites and fictitious water levels based on their respective open water site, for selected sites in Mahakam (min = minutes).

location	site	% change in duration of inundation	change in class	% change in duration of inundation	change in class
		*min per day*	-	*min per inundation*	-
Indonesia—Mahakam2	E1	-2.6	-	-10	from 1 to 2
	F1	-2.2	-	-0.2	-
	G2	-23	-	-8.5	from 1 to 2

At site F1, the water levels follow the tidal regime closely (Fig 7). These sites are located just outside of a restored shrimp pond (see Section “Study area description”). Almost no tailing is visible at the transition from high to low tide. There is some lagging of the outflow, which we believe is due to micro-topography, but for this site this has no influence on the resulting hydrological class (Table 6).

At site G2, tailing is obvious (Fig 7). During ebb, outflow is restricted, resulting in a longer duration of inundation. Inflow is also restricted at this site, but less than outflow. Without these restrictions, site G2 would have had shorter inundations, on average 23% shorter in *min per day* and 8.5% shorter in *min per inundation*, and a higher class, so drier conditions (Table 6).

### Comparison with classification based on elevation only

The Watson classification (Table 1) and the methodology proposed by [34] suggest that estimates of the hydrological conditions can be made based on information of elevation, without measuring water levels. To test this, we estimated the hydrological class for all sites based on elevation alone (by combining information from Tables 2 and 4). This results in reasonable classes for locations A and B in Vietnam (hydrological class in Table 7 comparable to vegetation class and to hydrological classes in Table 5), but unrealistically low classes for locations C–G in Mahakam. The estimated classes based on elevation alone for these sites are much lower than calculated using inundation characteristics (Table 5) or than expected based on vegetation (Table 7). If this method would be used in restoration projects for the disturbed sites (E–G), this would lead to planting the wrong species.

**Table 7:** Hydrological classification based on elevation only for all sites. Disturbed sites in Mahakam (E–G) compared to matching natural sites (C–D; based on elevation) and their vegetation class.

location	site	hydrological class based on elevation only	vegetation class	paired site	vegetation class of paired site
Can Gio	A1	2	2		
	A2	2*	2*		
	A3	3	2*		
	A4	3	3		
	A5	3	3		
	A6	3	4		
Ca Mau	B1	2	2*		
	B2	2	2*		
Mahakam	C1	2*	2*		
	C2	2*	2*/3		
	C3	1	2*		
	D1	1	2		
	D2	1	2/2*		
	D3	2	2*/3		
	D4	2*	4		
	E1	1	-	D1	2
	E2	1	-	C3	2*
	E3	2	-	D3	2*/3
	F1	1	2*	C3	2*
	F2	2	3	D3	2*/3
	G1	2	2*	D3	2*/3
	G2	1	-	D1	2
	G3	2	3	D3	2*/3

We, therefore, tested if an alternative approach, based on finding matching natural sites based on elevation, could give similar information as derived from water levels. We took elevation of the disturbed sites (E–G) from Table 4, found matching natural sites (C–D) based on elevation (Table 4) and extracted the vegetation classification for that site from Table 5. This resulted in a good match between observed and estimated vegetation for a number of sites (F1, F2, G1 and G3), indicating that planting species based on the matching natural site could be successful. For the obstructed sites (E1–E3 and G2), planting the species found in the matching natural sites could lead to success in sites E2 and E3, because their hydrological classes correspond to the vegetation classes of the natural site (Table 5). However, for sites with heavy tailing due to obstruction (E1 and G2), planting according to matching natural sites would result in failure because of too wet hydrological conditions. Mimicking the topography of the natural mangrove site in the disturbed site (as suggested by [34]) might solve this problem if it means that all obstructions are removed. Removing only the weir or breaching the dikes in one place is not sufficient to reduce duration of inundation enough for mangrove species to grow and survive, as was shown for site G2.

Major drawbacks of this alternative approach, however, are that location-specific effects such as micro-topography are not taken into account and that accurately measuring surface level elevation in mangrove forests is not straightforward. These uncertainties are further explored below in the Section “Sensitivity analysis”.

### Designing the restoration of appropriate hydrology

Based on the analysis done here, we could give the following management advice for restoration of the hydrologically disturbed sites in Mahakam (Indonesia):

- In sites with vegetation and matching hydrological conditions (sites F1, F2, G1, and G3), natural generation was possible or planting was done with the right species (unknowingly). In these sites there is no action needed; only monitoring whether vegetation stays healthy and natural regeneration takes place.
- In sites where the hydrological conditions are suitable but no vegetation is present (sites E2 and E3), natural regeneration is hampered by other factors than hydrology. When other factors like salinity and soil conditions are favourable, there probably are obstacles that impede propagules to reach the site. In sites E2 and E3 these obstacles are the shrimp farm dikes. A possible solution is to remove the dikes to allow for natural regeneration or to plant species belonging to the estimated hydrological class.
- In sites where the hydrological conditions are unsuitable and there is no vegetation (site E1 and G2), natural generation did not occur due to too wet conditions or *Rhizophora sp.* planting failed because of too wet conditions. The solution is to restore the hydrological conditions, then repeat the water level measurements and apply the classification again, and finally, allow for natural regeneration or plant the species belonging to the new hydrological class.

## Sensitivity analysis

### Uncertainties in hydrological variables

In this study, we aimed at a relatively simple but robust method to determine the hydrological conditions relevant for mangrove species. This method has uncertainties related to the measurement of water levels and the way water level measurements are converted into duration of inundation. The measurement equipment itself is very accurate, with an estimated accuracy of 0.05% for the pressure measurements which is translated into a field accuracy of 0.5–1 cm in our field setup [63], and the resolution of the measurements is high with a 5-minute interval. The estimation of surface level, however, could be prone to errors because it depends on expert judgement. For locations E–G in Indonesia surface level estimations by an independent expert were available [67], allowing for a sensitivity analysis. Application of the alternative surface levels in the calculation of inundation characteristics resulted in a 1.5% difference in duration of inundation in *min per day* and a 3.0% difference in *min per inundation*, on average for all sites. These minor differences in inundation characteristics did not result in changes in class. This analysis shows that the differences in inundation duration due to methodology are in the same order of magnitude as differences in inundation duration between mangrove sites and open water sites in unobstructed sites (e.g. F1; Table 6), whereas the difference is larger in obstructed sites (e.g. G2; Table 6). This means that the effect of obstruction on inundation duration is larger than the effect of uncertainties in the methodology.

Additionally, a longer time period (ca. 50 days, missing a period between neap tide and spring tide) of water level measurements was available for locations E–G in Indonesia. For the analysis in this paper we only used 30 days to match the monthly tidal cycle (Table 4), but the full period could be used to evaluate the sensitivity of the classification to the period of measurement. Inundation characteristics for the longer period had a 3.2% difference in duration of inundation in *min per day* and a 4.0% difference in *min per inundation*, on average for all sites. For most sites, inundations were shorter in the 50-day period compared to the 30-day period, because the 50-day period missed a wet period in the tidal cycle. This indicates that using measurement periods of a multitude of 30 days is best in regions with irregular tidal regime, but the error is relatively small when using a longer or shorter period. Only for site G2 the 50-day period resulted in a different (drier) hydrological class, because one of the inundation variables just crossed a class boundary.

### Uncertainties in elevation analysis

As mentioned in the “Results and Discussion” section, the estimation of hydrological conditions based on elevation alone has major uncertainties. Measuring surface level elevation in mangrove forests correctly is a huge challenge. In this study, we used water levels to determine elevation, because laser levelling proved to have major errors [40]. Our method, however, also has some assumptions that require discussion. For example, we used the average water levels measured by the open water diver to represent mean sea level. This was done out of necessity because for some of the locations there was no tidal station close by from which we could use sea level observations. Research [68] has shown that differences in water levels between open sea and river delta can be in the order of 0 cm [69] to 10–15 cm [70].

Additionally, because we measure water levels for a limited time (one lunar tidal cycle), seasonal differences in water levels are not taken into account. For Mahakam, this does not have large consequences because the seasonal variation in river discharge is minimal [56, 57]. For Can Gio, effects are also expected to be low because measurements were done in three months at the transition from dry to wet season (Table 4). In Ca Mau, measurements were done in one month in the wet season, with potentially high effects on river discharge and water levels [68], but no rivers with a large upstream catchment are present in Ca Mau. If seasonality and the influence of river discharge are high, the measured open water level can still be used but only as local reference level. Then it does not represent mean sea level. This makes it then very difficult to compare sites in different regions or to use the elevation in the classification of Tables 1 or 2.

The assumption that water levels are level in the open water and throughout the mangrove forest at maximum high tide is consistent with observations done by [69] and [66]. In their book [66], state that ‘that the water surface at high tide forms a horizontal surface throughout the area’, illustrating this point with observations of water levels in a mangrove forest in Japan. Contrastingly, in their hydrodynamical model, [71] modelled lower and delayed maximum water levels inside the mangrove swamp compared to the creek. These modelled lower maximum water levels have, as far as we know, never been confirmed by measurements. The delay between when maximum high tide is reached in the creek and in the mangrove forest is confirmed by observations [69] for a site in Australia, but in our study locations in Vietnam and Indonesia these delays are minimal (in the order of a few minutes). Theoretically, however, maximum water levels might be expected to vary within a mangrove forest, for example due to tidal asymmetry in flood and ebb velocities and durations [72–74].

These uncertainties are all the more reason not to use elevation as a proxy for hydrological conditions but to measure water levels over a lunar tidal cycle of 30 days, calculate duration of inundation, and estimate an accurate hydrological class. After pioneering work of [75], water levels in mangrove forests have scarcely been measured [76]. Elevation is almost always taken as proxy for inundation characteristics, but there is little quantification of whether that is acceptable [9]. Based on the results of this study, we argue for measuring of water levels in mangrove areas, as hydrology is an often disregarded but important factor for mangrove vegetation [9, 22] and its relationship with elevation is often more complex than initially thought.

The spatial representativity of the measurements done at a specific site is dependent on local circumstances. In areas with relatively uniform conditions, e.g. an extensive mudflat along a river (location A and D), spatial representativity can be large. But in most regions, and especially in restoration sites, hydrological conditions can change relatively quickly in space because of micro-topography and the presence of dikes and levees [39]. Therefore, multiple measurement sites should be selected within a restoration plot. Furthermore, visual inspection is always needed for site selection and interpretation of site measurements. For example a series of photos can be taken over one (or more) low and high tides to determine ponding areas or dry spots.

### Uncertainties in vegetation analysis

The agreement between hydrological class and vegetation class in the natural sites was not always perfect. It was higher for the regions in which the classification was developed (Can Gio), but this might also be due to the fact that the relatively old mangrove forests in Can Gio exhibited a well-developed mangrove distribution. In young mangrove areas, i.e. location B (Ca Mau), or areas with less well-developed distribution, i.e. location C (Mahakam), conditions are more variable and mangrove species more mixed.

In some natural sites (e.g. A6 in Can Gio and C3 in Indonesia; Table 5) we noticed that the vegetation class was higher than the hydrological class. This means that species grow at wetter than expected conditions. This is striking because the expectation is that species establish at wet conditions, the site becomes drier due to sediment accretion, and adult trees survive these drier conditions. This happened for example at site B1, which had a vegetation class lower than the hydrological class (Table 5).

The expected class was determined from vegetation inventories in the area directly surrounding the measurement site. At many sites, especially in Indonesia, a number of different mangrove species were present that represent a variety of classes. As mentioned in Section “Data and Methods” the most dominant species and the species with most juvenile trees were used in the determination of the vegetation class. However, the co-existence of species within different hydrological classes shows that there is some overlap between the classes [15]. The classification should, therefore, not be applied in a very rigid way.

The fact that some sites had suitable hydrological conditions but no vegetation shows that hydrology is not the only factor in determining mangrove species. [77] even critiques the focus on hydrology alone in explaining mangrove species distribution. Soil factors such as soil structure, salinity, and redox state, play an important role [13] and nutrient and light conditions influence natural regeneration of mangroves [15]. [78] for example showed that many mangrove restoration projects fail because of acid sulfate soils. In a study in Indonesia [79], free tidal inundation at abandoned pond sites improved the sediment quality. This shows that salinity and soil conditions are linked to the hydrology and might improve when hydrology is restored. Some authors [12] point out that morphodynamic requirements should also not be neglected in mangrove restoration projects.

In regions with different mangrove species and different distribution patterns, e.g. South-America and Africa, the classification used here can still be applied. But before applying it to mangrove restoration projects, the local vegetation classes (last column in Table 2) need to be determined first in a natural mangrove stand, as was done for example by [80] for the ‘New World’ mangroves using Watson’s classification [31].

## Recommendations for mangrove restoration projects

Based on the results of this study, we propose the following methodology to determine hydrological suitability for mangrove restoration:

- Inspect the location for which restoration is desired and select measurement sites.
- Place water level loggers in a tube below surface level (no need for a complicated measurement set up or measurement of surface level).
- Measure water levels for one or multiple lunar tidal cycles (period of 30 days or a multitude of 30 days). Make sure visual inspection of in and outflow of water is carried out at different stages during the measurement period.
- Calculate inundation characteristics, duration of inundation in *min per day* and *min per inundation* with a robust analysis method as proposed here (Section “Data and Methods”). Use the hydrological classification of Table 2 to determine the hydrological class of the measurement sites.
- Determine whether hydrological restoration is needed (removal of dikes and weirs), whether natural regeneration is possible or planting is needed, and which species are most suitable for which site.

We have shown here that using only tidal information or water levels measured in open water close to the site, does not give sufficient information to characterise the hydrological situation of the restoration site (see Section “Results and Discussion”). Additionally, using elevation alone can only be successful if i) elevation is measured accurately, ii) matching natural sites can be found, and iii) hydrological restoration is done to match the topography of the natural site. Hydrological restoration of shrimp ponds in practice means more than dismantling of weirs; it should include the removal of dikes. This restores the hydrological conditions, reducing the duration of inundation, but it also allows for better distribution of the mangrove propagules, making natural regeneration more likely [12, 24]. Natural regeneration can be enhanced by planting mangroves [81], if the right species are chosen for the right location [9].

The methodology proposed above still needs to be tested by applying the hydrological classification in a mangrove restoration project and monitoring the success (or failure) of the project over time. Up to date there has been little evaluation of the effectiveness of the methodologies used in mangrove restoration projects [28, 82].

## Conclusions

The approach presented in this study provides a simple but robust methodology for using local water level data in mangrove restoration projects. A previously developed hydrological classification for mangroves was applied to 23 measurement sites in seven locations in Vietnam and Indonesia. For the natural sites, a good match was found between the hydrological class (based on two variables of duration of inundation) and expected class based on vegetation. For the hydrologically disturbed sites, the classification indicated whether hydrological conditions were or could be suitable for mangrove restoration. From the eight hydrologically disturbed sites evaluated in this study, three already had the mangrove vegetation matching the hydrological conditions (either due to planting or naturally), two did not have any vegetation but hydrological conditions were suitable for a range of mangrove species, and two had too wet conditions for mangrove growth due to obstructions that caused 3 to 23% longer inundations and a lower hydrological class, representing wetter conditions. For these last two sites the hydrology needs to be restored by removing dikes before natural regeneration or planting is possible.

This analysis has shown that a hydrological classification based on measured water levels can be a powerful tool in mangrove restoration, despite uncertainties in the calculation of the hydrological variables and vegetation. The alternative approach of estimating suitable mangrove species by matching the restoration site to a natural site based on elevation [34] can give comparable results, if the topography and hydrology are completely restored. However, elevation is most accurately estimated using water levels. Therefore, we advocate measurement of water levels for a minimal period of a lunar tidal cycle (30 days) and application of the hydrological classification based on duration of inundation. We note that the classification should not be applied too strictly; users should bare in mind the spatial variability of inundation, the range of suitable hydrological conditions for mangrove species, and the fact that factors other than hydrology play a role as well.

This paper provides information on practical management implications of research in mangrove areas, which is urgently needed according to [83] and [27]. Application of the methodology presented here will hopefully end the practice of planting in mudflats [11, 23, 84] and in abandoned shrimp farms with dikes intact, and will result in more hydrologically-sound mangrove restoration projects.

## References

[1] Zedler JB, Kercher S. Wetland Resources: Status, Trends, Ecosystem Services, and Restorability. Annual Review of Environment and Resources. 2005;30(1):39–74. 10.1146/annurev.energy.30.050504.144248

[2] Lee SY, Primavera JH, Dahdouh-Guebas F, McKee K, Bosire JO, Cannicci S, et al Ecological role and services of tropical mangrove ecosystems: a reassessment. Global Ecology and Biogeography. 2014;23(7):726–743. 10.1111/geb.12155

[3] Murdiyarso D, Purbopuspito J, Kauffman JB, Warren MW, Sasmito SD, Donato DC, et al The potential of Indonesian mangrove forests for global climate change mitigation. Nature Clim Change. 2015;5:1089–1092. 10.1038/nclimate2734

[4] Valiela I, Bowen JL, York JK. Mangrove Forests: One of the World’s Threatened Major Tropical Environments. Bioscience. 2001;51(10):807–815. 10.1641/0006-3568(2001)051[0807:MFOOTW]2.0.CO;2

[5] Alongi DM. Present state and future of the world’s mangrove forests. Environmental conservation. 2002;29(03):331–349. 10.1017/S0376892902000231

[6] Polidoro BA, Carpenter KE, Collins L, Duke NC, Ellison AM, Ellison JC, et al The loss of species: mangrove extinction risk and geographic areas of global concern. PLoS One. 2010;5(4):e10095 10.1371/journal.pone.0010095 20386710 PMC2851656

[7] Friess DA, Webb EL. Variability in mangrove change estimates and implications for the assessment of ecosystem service provision. Global ecology and biogeography. 2014;23(7):715–725. 10.1111/geb.12140

[8] Richards DR, Friess DA. Rates and drivers of mangrove deforestation in Southeast Asia, 2000–2012. Proceedings of the National Academy of Sciences. 2015;. 10.1073/pnas.1510272113 PMC4720307 26712025

[9] Lewis RR III. Ecological engineering for successful management and restoration of mangrove forests. Ecological Engineering. 2005;24(4):403–418. 10.1016/j.ecoleng.2004.10.003

[10] Primavera JH, Esteban JMA. A review of mangrove rehabilitation in the Philippines: successes, failures and future prospects. Wetlands Ecology and Management. 2008;16(5):345–358. 10.1007/s11273-008-9101-y

[11] Samson MS, Rollon RN. Growth Performance of Planted Mangroves in the Philippines: Revisiting Forest Management Strategies. AMBIO: A Journal of the Human Environment. 2008;37(4):234–240. 10.1579/0044-7447(2008)37[234:GPOPMI]2.0.CO;2 18686501

[12] Winterwerp JC, Erftemeijer PLA, Suryadiputra N, van Eijk P, Zhang L. Defining Eco-Morphodynamic Requirements for Rehabilitating Eroding Mangrove-Mud Coasts. Wetlands. 2013;33(3):515–526. 10.1007/s13157-013-0409-x

[13] Matthijs S, Tack J, van Speybroeck D, Koedam N. Mangrove species zonation and soil redox state, sulphide concentration and salinity in Gazi Bay (Kenya), a preliminary study. Mangroves and Salt Marshes. 1999;3(4):243–249. 10.1023/A:1009971023277

[14] Ellison AM, Mukherjee BB, Karim A. Testing patterns of zonation in mangroves: scale dependence and environmental correlates in the Sundarbans of Bangladesh. Journal of Ecology. 2000;88(5):813–824. 10.1046/j.1365-2745.2000.00500.x

[15] Krauss KW, Lovelock CE, McKee KL, López-Hoffman L, Ewe SML, Sousa WP. Environmental drivers in mangrove establishment and early development: A review. Aquatic Botany. 2008;89(2):105–127. 10.1016/j.aquabot.2007.12.014

[16] Gopal B. Future of wetlands in tropical and subtropical Asia, especially in the face of climate change. Aquatic Sciences. 2013;75(1):39–61. 10.1007/s00027-011-0247-y

[17] Field C, Osborn J, Hoffman L, Polsenberg J, Ackerly D, Berry J, et al Mangrove biodiversity and ecosystem function. Global Ecology & Biogeography Letters. 1998;7(1):3–14. 10.2307/2997693

[18] Elster C. Reasons for reforestation success and failure with three mangrove species in Colombia. Forest Ecology and Management. 2000;131(1):201–214. 10.1016/S0378-1127(99)00214-5

[19] Gilman E, Ellison J. Efficacy of alternative low-cost approaches to mangrove restoration, American Samoa. Estuaries and Coasts. 2007;30(4):641–651. 10.1007/BF02841961

[20] Bosire JO, Dahdouh-Guebas F, Walton M, Crona BI, Lewis RR III, Field C, et al Functionality of restored mangroves: A review. Aquatic Botany. 2008;89(2):251–259. 10.1016/j.aquabot.2008.03.010

[21] Zaldívar-Jiménez MA, Herrera-Silveira JA, Teutli-Hernández C, Comín FA, Andrade JL, Molina CC, et al Conceptual framework for mangrove restoration in the Yucatán Peninsula. Ecological Restoration. 2010;28(3):333–342. 10.3368/er.28.3.333

[22] Friess DA, Krauss KW, Horstman EM, Balke T, Bouma TJ, Galli D, et al Are all intertidal wetlands naturally created equal? Bottlenecks, thresholds and knowledge gaps to mangrove and saltmarsh ecosystems. Biological Reviews. 2012;87(2):346–366. 10.1111/j.1469-185X.2011.00198.x 21923637

[23] Erftemeijer PLA, Lewis III RR. Planting mangroves on intertidal mudflats: habitat restoration or habitat conversion. In: Proceedings of the ECOTONE VIII seminar enhancing coastal ecosystems restoration for the 21st century, Ranong, Thailand; 1999. p. 23–28.

[24] Brockmeyer RE Jr, Rey JR, Virnstein RW, Gilmore RG, Earnest L. Rehabilitation of impounded estuarine wetlands by hydrologic reconnection to the Indian River Lagoon, Florida (USA). Wetlands Ecology and Management. 1996;4(2):93–109. 10.1007/BF01876231

[25] Stevenson NJ, Lewis RR III, Burbridge PR. Disused shrimp ponds and mangrove rehabilitation In: An international perspective on wetland rehabilitation. Springer; 1999 p. 277–297.

[26] Matsui N, Suekuni J, Nogami M, Havanond S, Salikul P. Mangrove rehabilitation dynamics and soil organic carbon changes as a result of full hydraulic restoration and re-grading of a previously intensively managed shrimp pond. Wetlands Ecology and Management. 2010;18(2):233–242. 10.1007/s11273-009-9162-6

[27] Van Bochove J, Sullivan E, Nakamura Te, editors. The Importance of Mangroves to People: A Call to Action. UNEP, United Nations Environment Programme World Conservation Monitoring Centre, Cambridge. 128 pp.; 2014.

[28] Lewis RR III. Methods and criteria for successful mangrove forest restoration in Coastal Wetlands: An Integrated Ecosystem Approach Perillo G M E, Wolanski E, Cahoon D R, and Brinson M M (eds) Elsevier, Amsterdam, the Netherlands 2009;p. 787–800.

[29] Balke T, Bouma TJ, Horstman EM, Webb EL, Erftemeijer PLA, Herman PMJ. Windows of opportunity: thresholds to mangrove seedling establishment on tidal flats. Marine Ecology Progress Series. 2011;440 10.3354/meps09364

[30] Crase B, Liedloff A, Vesk PA, Burgman MA, Wintle BA. Hydroperiod is the main driver of the spatial pattern of dominance in mangrove communities. Global Ecology and Biogeography. 2013;22(7):806–817. 10.1111/geb.12063

[31] Watson JG. Mangrove forests of the Malay Peninsula. 6 Fraser & Neave; 1928.

[32] Tomlinson P. The botany of mangroves Cambridge tropical biology series. Cambridge University Press, Cambridge; 1986.

[33] Wolanski E. Hydrodynamics of mangrove swamps and their coastal waters. Hydrobiologia. 1992;247(1-3):141–161. 10.1007/BF00008214

[34] Lewis RR III, Quarto A, Enright J, Corets E, Primavera J, Ravishankar DSO T, et al‥ Five Steps to Successful Ecological Restoration of Mangroves; 2006.

[35] Marchand M. Mangrove restoration in Vietnam: Key considerations and a practical guide. Deltares (available at: http://repository.tudelft.nl/view/ir/uuid:98b5ba43-1452-4631-81dc-ad043ef3992c/, last access: 05-07-2015); 2008.

[36] Punwong P, Marchant R, Selby K. Holocene mangrove dynamics from Unguja Ukuu, Zanzibar. Quaternary International. 2013;298:4–19. 10.1016/j.quaint.2013.02.007

[37] Punwong P, Marchant R, Selby K. Holocene mangrove dynamics in Makoba Bay, Zanzibar. Palaeogeography, Palaeoclimatology, Palaeoecology. 2013;379:54–67. 10.1016/j.palaeo.2013.04.004

[38] Di Nitto D, Neukermans G, Koedam N, Defever H, Pattyn F, Kairo JG, et al Mangroves facing climate change: landward migration potential in response to projected scenarios of sea level rise. Biogeosciences. 2014;11(3):857–871. 10.5194/bg-11-857-2014

[39] Van Loon AF, Dijksma R, Van Mensvoort MEF. Hydrological classification in mangrove areas: A case study in Can Gio, Vietnam. Aquatic Botany. 2007;87(1):80–82. 10.1016/j.aquabot.2007.02.001

[40] Te Brake B, Van Huijgevoort MHJ. Hydrological characterization of mangrove forests in Can Gio and Ca Mau, Vietnam. MSc. Thesis, Department of Environmental Sciences, Wageningen University, The Netherlands (available on: http://www.slideshare.net/BramteBrake/te-brake-and-van-huijgevoort-2008-hydrological-classification-of-mangrove-forests-in-can-gio-and-ca-mau-vietnam, last access: 05-07-2015); 2008.

[41] Dijksma R, Van Loon AF, Van Mensvoort MEF, Van Huijgevoort MHJ, Te Brake B. 28 An Extended Hydrological Classification for Mangrove Rehabilitation Projects: a Case Study in Vietnam. Tropical Deltas and Coastal Zones: Food Production, Communities and Environment at the Land-Water Interface. 2010;9:384.

[42] Tuan VQ, Kuenzer C, editors. Can Gio Mangrove Biosphere Reserve Evaluation 2012: Current Status, Dynamics, and Ecosystem Services. IUCN, Hanoi, Viet Nam 102 pp.; 2012.

[43] Binh TNKD, Vromant N, Hung NT, Hens L, Boon EK. Land cover changes between 1968 and 2003 in Cai Nuoc, Ca Mau peninsula, Vietnam. Environment, Development and Sustainability. 2005;7(4):519–536. 10.1007/s10668-004-6001-z

[44] Tutiempo Network SL. http://en.tutiempo.net/climate/2009/ws-966070.html. Retrieved November 20, 2015. 2009;.

[45] Tutiempo Network SL. http://en.tutiempo.net/climate/2011/ws-966070.html. Retrieved March 26, 2012. 2011;.

[46] Hong PN, San HT. Mangroves of Vietnam. Bangkok, Thailand: IUCN—The World Conservation Union; 1993.

[47] Vietnam National Committee. Can Gio Mangrove Biosphere Reserve. Draft Biosphere Reserve Nomination Form. Ho Chi Minh City, Vietnam: Vietnam National Committee; 1998.

[48] Tri N, Hong P, Cuc L. Can Gio Mangrove Biosphere Reserve Ho Chi Minh City. Hanoi, Vietnam. 2000;.

[49] Miyagi T, Nam VN, Sinh LV, Kainuma M, Saitoh A, Hayashi K, et al. Further Study on the Mangrove Recovery Processes in Can Gio, Viet Nam. In: Chan HT, Cohen M, editors. Studies in Can Gio Mangrove Biosphere Reserve, Ho Chi Minh City, Viet Nam. vol. No. 6 of ISME Mangrove Ecosystems Technical Reports. Tohoku Gakuin University; Can Gio Mangrove Protection Forest Management Board; International Society for Mangrove Ecosystems (ISME); 2013.

[50] Chien NV, Nguyen LD, Viet PB. A Case Study of Can Gio Mangrove System, Vietnam. Study on Coastal Zone Environment Management with Emphasis on Mangrove Ecosystem to Assist in Poverty Alleviation Bangkok, Thailand, ESCAP Regional Space Applications Programme for Sustainable Development (RESAP), Economic and Social Commission for Asia and the Pacific. 2003;.

[51] Tuan LD, Oahn TTK, Thanh CV, Qui ND. Can Gio Mangrove Biosphere Reserve. Oxfam America; 2002.

[52] Vogt J, Kautz M, Herazo MLF, Triet T, Walther D, Saint-Paul U, et al Do canopy disturbances drive forest plantations into more natural conditions? A case study from Can Gio Biosphere Reserve, Viet Nam. Global and Planetary Change. 2013;110, Part B:249–258. 10.1016/j.gloplacha.2011.09.002

[53] Tong PHS, Auda Y, Populus J, Aizpuru M, Habshi AA, Blasco F. Assessment from space of mangroves evolution in the Mekong Delta, in relation to extensive shrimp farming. International Journal of Remote Sensing. 2004;25(21):4795–4812. 10.1080/01431160412331270858

[54] Van TT, Wilson N, Thanh-Tung H, Quisthoudt K, Quang-Minh V, Xuan-Tuan L, et al Changes in mangrove vegetation area and character in a war and land use change affected region of Vietnam (Mui Ca Mau) over six decades. Acta Oecologica. 2015;63:71–81. 10.1016/j.actao.2014.11.007

[55] Gowing J, Tuong T, Hoanh CT, Khiem N. Social and environmental impact of rapid change in the coastal zone of Vietnam: an assessment of sustainability issues. Environment and Livelihoods in Tropical Coastal Zones: Managing Agriculture–Fishery–Aquaculture Conflicts CAB International, Wallingford, UK. 2006;p. 48–60.

[56] Storms JEA, Hoogendoorn RM, Dam RAC, Hoitink AJF, Kroonenberg SB. Late-Holocene evolution of the Mahakam delta, East Kalimantan, Indonesia. Sedimentary Geology. 2005;180(3-4):149–166. 10.1016/j.sedgeo.2005.08.003

[57] Sassi MG, Hoitink AJF, de Brye B, Deleersnijder E. Downstream hydraulic geometry of a tidally influenced river delta. Journal of Geophysical Research: Earth Surface. 2012;117(F4). F04022 10.1029/2012JF002448

[58] Mandang I, Yanagi T. Tide and Tidal Current in the Mahakam Estuary, East Kalimantan, Indonesia. Coastal marine science. 2008;32(1):1–8.

[59] Gastaldo RA. Peat or no peat: Why do the Rajang and Mahakam Deltas differ? International Journal of Coal Geology. 2010;83:162–172. 10.1016/j.coal.2010.01.005

[60] Hall D. Explaining the Diversity of Southeast Asian Shrimp Aquaculture. Journal of Agrarian Change. 2004;4(3):315–335. 10.1111/j.1471-0366.2004.00081.x

[61] Persoon GA, Simarmata R. Undoing’marginality’: The islands of the Mahakam Delta, East Kalimantan (Indonesia). Journal of Marine and Island Cultures. 2014;3(2):43–53. 10.1016/j.imic.2014.11.002

[62] Sidik AS. The Changes of Mangrove Ecosystem in Mahakam Delta, Indonesia: A complex social-environmental pattern of linkages in resources utilization. Paper presented at The South China Sea Conference 2008 The South China Sea: Sustaining Ocean Productivities, Maritime Communities and the Climate Kuantan, Malaysia, 25–29 November 2008. 2008;.

[63] Eijkelkamp. Ground water monitoring and communication solutions: Diver / e-SENSE. Giesbeek, the Netherlands. http://diver-water-level-logger.com/ [last access: 30-7-2015]; 2014.

[64] Nam VN, Thuy NS. Identifying Plants In Mangroves By Pictures. TP Ho Chi Minh.; 1999.

[65] Krauss KW, Doyle TW, Twilley RR, Rivera-Monroy VH, Sullivan JK. Evaluating the relative contributions of hydroperiod and soil fertility on growth of south Florida mangroves. Hydrobiologia. 2006;569(1):311–324. 10.1007/s10750-006-0139-7

[66] Mazda Y, Wolanski E, Ridd P. The role of physical processes in mangrove environments: manual for the preservation and utilization of mangrove ecosystems. 2007;.

[67] Van der Lely C. A Tool Towards Mangrove Restoration: Investigating the hydrology of abandoned shrimp ponds in South East Asia. MSc. Thesis, Department of Environmental Sciences, Wageningen University, The Netherlands; 2012.

[68] Wassmann R, Hien N, Hoanh C, Tuong T. Sea Level Rise Affecting the Vietnamese Mekong Delta: Water Elevation in the Flood Season and Implications for Rice Production. Climatic Change. 2004;66(1-2):89–107. 10.1023/B:CLIM.0000043144.69736.b7

[69] Aucan J, Ridd PV. Tidal asymmetry in creeks surrounded by saltflats and mangroves with small swamp slopes. Wetlands Ecology and Management. 2000;8(4):223–232. 10.1023/A:1008459814925

[70] Horstman EM, Dohmen-Janssen CM, Hulscher SJMH. Flow routing in mangrove forests: A field study in Trang province, Thailand. Continental Shelf Research. 2013;71:52–67. 10.1016/j.csr.2013.10.002

[71] Mazda Y, Kanazawa N, Wolanski E. Tidal asymmetry in mangrove creeks In: Wong YS, Tam NY, editors. Asia-Pacific Symposium on Mangrove Ecosystems. vol. 106 of Developments in Hydrobiology. Springer Netherlands; 1995 p. 51–58.

[72] Wolanski E, Jones M, Bunt J. Hydrodynamics of a tidal creek-mangrove swamp system. Mar Freshwater Res. 1980 1;31(4):431–450. 10.1071/MF9800431

[73] Moore RD, Wolf J, Souza AJ, Flint SS. Morphological evolution of the Dee Estuary, Eastern Irish Sea, UK: A tidal asymmetry approach. Geomorphology. 2009;103(4):588–596. 10.1016/j.geomorph.2008.08.003

[74] Horstman EM, Dohmen-Janssen CM, Bouma TJ, Hulscher SJMH. Tidal-scale flow routing and sedimentation in mangrove forests: Combining field data and numerical modelling. Geomorphology. 2015;228:244–262. 10.1016/j.geomorph.2014.08.011

[75] Wolanski E, Mazda Y, Ridd P. Mangrove hydrodynamics. Coastal and Estuarine Studies. 1993;p. 43–43.

[76] Mazda Y, Kobashi D, Okada S. Tidal-Scale Hydrodynamics within Mangrove Swamps. Wetlands Ecology and Management. 2005;13(6):647–655. 10.1007/s11273-005-0613-4

[77] Clarke PJ. Seeking global generality: a critique for mangrove modellers. Mar Freshwater Res. 2014;65(10):930–933. 10.1071/MF13326

[78] Dent D. Reclamation of acid sulphate soils In: Soil restoration. Springer; 1992 p. 79–122.

[79] Isyrini R, Gust D, Williamson I, Scharaschkin T, Noor A. Natural improvements of geochemical conditions of acid sulfate soils caused by free tidal inundation and its effects on the mangrove seedlings. In: The Asian Conference on Sustainability, Energy & the Environment: Official Conference Proceedings 2012. The International Academic Forum (IAFOR); 2012. p. 361–367.

[80] Chapman VJ. Mangrove vegetation. Vaduz: J Cramer. 1976;581.

[81] Bosire JO, Dahdouh-Guebas F, Kairo JG, Koedam N. Colonization of non-planted mangrove species into restored mangrove stands in Gazi Bay, Kenya. Aquatic Botany. 2003;76(4):267–279. 10.1016/S0304-3770(03)00054-8

[82] Andradi-Brown DA, Howe C, Mace GM, Knight AT. Do mangrove forest restoration or rehabilitation activities return biodiversity to pre-impact levels? Environmental Evidence. 2013;2(1):20 10.1186/2047-2382-2-20

[83] Kairo JG, Dahdouh-Guebas F, Bosire J, Koedam N. Restoration and management of mangrove systems– a lesson for and from the East African region. South African Journal of Botany. 2001;67(3):383–389. 10.1016/S0254-6299(15)31153-4

[84] Agoramoorthy G. Planting mangroves in mudflats: is it the way of the world? Environmental science & technology. 2012;46(7):3625–3626. 10.1021/es300923j 22428528

